# Weighing in on heart failure: the potential impact of bariatric surgery

**DOI:** 10.1007/s10741-021-10078-w

**Published:** 2021-01-25

**Authors:** Tanuka Datta, Andrew J. Lee, Rachel Cain, Melissa McCarey, David J. Whellan

**Affiliations:** 1 Department of Internal Medicine, Division of Cardiology, Thomas Jefferson University, Philadelphia, PA USA; 2 Sidney Kimmel Medical College, Thomas Jefferson University, Philadelphia, PA USA

**Keywords:** Bariatric surgery, Heart failure, Cardiovascular disease, Weight loss, Decreased ventricular function, Cardiomyopathy, Metabolic syndrome, Weight loss

## Abstract

Obesity is a growing worldwide epidemic with significant economic burden that carries with it impacts on every physiologic system including the cardiovascular system. Specifically, the risk of heart failure has been shown to increase dramatically in obese individuals. The purpose of this review is to provide background on the individual burdens of heart failure and obesity, followed by exploring proposed physiologic mechanisms that interconnect these conditions, and furthermore introduce treatment strategies for weight loss focusing on bariatric surgery. Review of the existing literature on patients with obesity and heart failure who have undergone bariatric surgery is presented, compared, and contrasted.

## Introduction

Obesity is a growing worldwide epidemic at the level of both being a major health problem and a heavy economic burden. The disease carries with it impacts on metabolism that encompass almost every physiologic system, including the cardiovascular system. In fact, the risk of heart failure (HF) increases by 30–100% in obese individuals. Furthermore, obesity is an independent risk factor in the progression of heart failure ranging from effects on diastolic dysfunction to end stage left ventricular dilation with reduced systolic function [1]. Weight loss achieved from bariatric surgery has been recognized as a potential modality to improve such cardiac remodeling. Proposed mechanisms range from a mechanical decrease in cardiac workload to cellular phenomenon related to hyperleptinemia, reduced inflammation, and improved metabolism [2, 3]. As heart failure affects 6.5 million adults in the USA [4], with a significant subset of this population having comorbid obesity, the purpose of this review is to explore the relationship between heart failure and obesity with the goal of establishing the potential positive impact of bariatric surgery on heart failure.

We aim to first provide background on the individual burdens of heart failure and obesity, followed by exploring some of the proposed physiologic mechanisms that interconnect these conditions. We then introduce treatment strategies in weight loss, focusing on bariatric surgery, including general pre-surgical care and post-operative outcomes. Finally, we present a review of the existing literature on patients with obesity and heart failure having undergone bariatric surgery, ending with limitations of the existing studies and the future directions that lay ahead in this realm.

## The burden of heart failure

Heart failure is a substantial public health issue affecting about 6.5 million adults in the USA [4]. It is estimated that by 2030, this number will increase to over 8.5 million [5]. It is a significant cause of morbidity and mortality with symptoms that are often rapidly progressive with considerable impacts on quality of life and a terminal disease phase that is known to be refractory to treatment [5, 6].

Heart failure carries a high economic burden that is bound to increase without meaningful intervention [5]. The annual direct cost for heart failure in the USA has been calculated to be as high as $115 billion or about 3.3% of the nation’s healthcare spending [7, 8]. A bulk of the cost attributed to heart failure relates to hospitalizations [5]. While the number of primary heart failure hospitalizations in the USA has slightly decreased over the years (1.1 million in 2001 to 1.0 million in 2009), secondary heart failure hospitalizations have increased over the same period of time (2.7 million in 2001 to 3.1 million in 2009) [6].

The general management of heart failure revolves around treatment of its underlying causes (e.g., ischemic heart disease, cardiomyopathy) as well as any associated conditions (e.g., hypertension, diabetes mellitus) [9]. Interventions range from relatively unaggressive treatments, such as lifestyle modification and pharmacologic therapy, to more invasive ones, such as device therapy and cardiac transplantation [10]. Pharmacologic strategies vary by patient but center largely on control of comorbidities such as hypertension, hyperlipidemia, and other conditions associated with heart failure. Adequate treatment of comorbidities promotes weight loss, which in turn is protective from progressing to worsening heart failure.

## The burden of obesity

Obesity is a complex disease that has grown in the USA as a major health problem [13]. The prevalence of adult obesity has been on the rise since the 1970s and is currently estimated to be about 40% [14, 15]. If this trend continues, the prevalence of obesity in the USA may climb to over 50% by 2030 [16].

A higher prevalence of obesity has and will come with an increasing prevalence of several diseases linked to obesity, many of which have significant influence on morbidity and mortality [17]. These comorbidities include a host of cardiovascular diseases, type 2 diabetes mellitus, malignancies, obstructive sleep apnea, and several other debilitating conditions [18]. The problem starts young; weight gain in youth is a strong marker for the development of hypertension in adulthood. What is more, hypertension occurs 6 times more frequently in obese men than in lean men. In fact, it has been shown that a 10-kg higher body weight correlates with a 3-mm Hg higher systolic blood pressure and a 2.3-mm Hg higher diastolic blood pressure. Ultimately, this accounts for up to a 12% increased risk of coronary heart disease and a 24% increased risk of stroke [5]. Patients with obesity also have a higher likelihood of developing atrial fibrillation—for a multitude of reasons, but structurally in part due to the display of left atrial enlargement [20]. Lastly, a number of malignancies, including esophageal adenocarcinoma, colorectal cancer, and pancreatic cancer, have been associated with obesity [22].

In addition to the litany of health consequences attributable to obesity, the economic burden of obesity is substantial as well [23]. A 27% rise in inflation-adjusted per capita healthcare spending between 1987 and 2001 was attributable to increases in the proportion of and spending on obese individuals compared with those of normal weight [24]. Not to mention, comorbid diseases, such as diabetes and cardiovascular disease, have also had significant effects on healthcare spending [25]. Additionally, obesity has a negative impact on economic productivity, and its effects on metrics such as workplace absenteeism and disability payments have been widely studied [26]. In the USA, early death attributable to obesity creates an estimated total lost productivity cost of about $625 per obese person, or over $30 billion nationally [26].

## Obesity in heart failure

There is an association between heart failure and obesity. For every increase in BMI of 1, the risk of heart failure increases by 5% in men and 7% in women [21]. One of the main comorbidities of obesity is heart failure, with obese individuals having double the risk of heart failure as compared with individuals with normal weight [21]. In fact, several studies show that obesity is a risk factor for morbidity and mortality in heart disease and almost doubles the risk of developing HF [29]. Obesity not only leads to the increase in incidence of HF but evidence suggests that there is also a dose-effect of BMI and rates of hospitalization for HF exacerbations [21, 30–34].

The underlying mechanism of cardiac dysfunction in patients with obesity is multifactorial and complex but is believed to be a result of several interconnecting systems including hyperleptinemia and ventricular remodeling.

## Hyperleptinemia

Adipose tissue in patients with obesity plays a very important endocrinologic role in contributing to the pathophysiology of cardiomyopathy as a result of obesity. On a cellular level, in a cardiac myocyte, the intracellular protein, signal transducer, and activator of transcription 3 (STAT3) exists to regulate many cellular processes including promotion of cardiac muscle differentiation and extracellular matrix homeostasis. STAT3 activation has been shown to be beneficial for the heart and play a role in the protection of ischemic heart disease. Among many extracellular ligands, leptin is one that promotes STAT3 signaling. At physiologic levels, this activation is beneficial; however, at supraphysiologic levels, leptin activation of STAT3 can lead to left ventricular hypertrophy [6]. While leptin is secreted from adipose tissue, cardiac cells themselves can produce leptin and appear to synthesize more leptin in patients with heart failure, leading to the increase in local as well as systemic leptin [35]. Currently, the only known causes of hyperleptinemia are overnutrition and diet-induced obesity [36, 37]. Interestingly, the high levels of leptin and leptin resistance have been linked to the development of cardiovascular disease [38]. As described above, high leptin levels seem to correlate with left ventricular wall mass or wall thickness, independent of high blood pressure, indicating leptin may play a central role in the development of cardiomyopathy [39]. Despite leptin resistance in the hypothalamus, cardiac cells are found to continue to respond to leptin in chronically high states, which is believed to trigger the development of cardiac hypertrophy in addition to maladaptive cardiac remodeling by the inability to activate the STAT-3 dependent cardioprotective pathways [40].

## Ventricular remodeling

As a result of the accumulation of adipose tissue and increased body mass, patients with obesity have a higher blood volume that requires higher cardiac output, which is achieved by increasing stroke volume, all of which leads to higher cardiac workload [19]. These hemodynamic changes lead to increased left ventricular size, wall stress, and subsequent eccentric left ventricular hypertrophy, subsequently predisposing to left ventricular failure [2]. As these myocardial changes take place, the right ventricle hypertrophies in response to the increased pulmonary venous pressures leading to pulmonary hypertension as a result of left ventricular failure. As obstructive sleep apnea is often a co-existent condition, hypoxia and acidosis develop as well further perpetuating these physiologic changes [29]. Several studies have found that after substantial weight loss, parameters of the LV remodeling and maladaptive hypertrophy are reversed, such as LV dilatation, hypertrophy, and mass [29].

## The other end of the spectrum

As we have thus far focused on the mechanisms and effects of obesity, it is worthwhile to briefly also explore the other end of the spectrum to provide a complete picture: low BMI and cachexia as it relates to heart failure. While some have proposed the theory of an “obesity paraodox” arguing that higher BMI’s are protective in the heart failure population, it is more accurate to perceive the relationship of BMI with outcomes in heart failure patients as a “J-shaped curve” [30]. Hence, one should keep in mind that although obesity is driving one end of poor outcomes in heart failure patients, cardiac cachexia and low BMI are equally as detrimental. Severe muscle wasting is frequently associated with advanced heart failure, with systemic inflammation as well as tumor necrosis factor playing a key role in the process. Many chronic disease states such as cancer trigger a similar progression of malnutrition and poor nutrient intake; however, specifically in the heart failure patient, this physiologic state is referred to as cardiac cachexia and is associated with not only a decreased quality of life but also poor survival [31]. Some of the mechanisms driving this severe state of malnutrition include anorexia from cytokine production, gut edema, and aversion to food intake due to fatigue and increased work of breathing [32]. These mechanisms are often very difficult if not impossible to reverse, and once a patient displays this state of being, palliation and end of life care are recommended.

## Treatment strategies for weight loss

Management of obesity revolves around weight loss. The approach to weight loss varies by BMI range, with more aggressive therapies being recommended for patients with higher BMI’s. Interventions range from lifestyle modifications to pharmacologic therapy to bariatric surgery. While there are a variety of diets available with different macronutrient compositions, diet adherence itself is one of the strongest predictors of weight loss [7]. Medications are also an option for those who need additional assistance. While specific pharmacologic approaches vary by patient, a meta-analysis comparing weight loss among obesity drug treatments found that orlistat, lorcaserin, naltrexone-bupropion, phentermine-topiramate, and liraglutide, compared with placebo, were associated with at least 5% weight loss within 52 weeks [8].

## Bariatric surgery

**Fig. 1:**
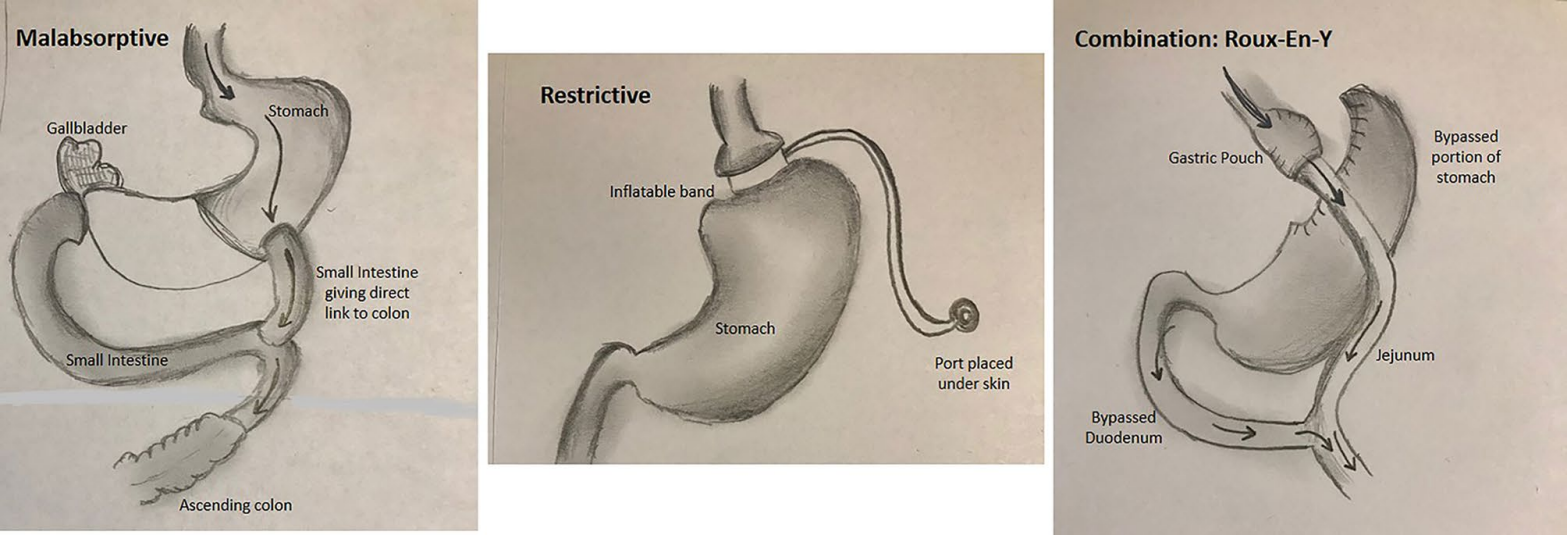
Several different bariatric surgery procedures

Bariatric surgery is a widely used and highly successful weight-loss strategy that has shown to be effective in lowering BMI and body weight in obese individuals. It is the single most effective method to achieve substantial weight loss [9]. Clinically, bariatric surgery and subsequent weight loss have been shown to significantly improve mortality and morbidity from heart disease in patients with and without established cardiac pathology [10, 11]. Current guidelines for consideration of bariatric surgery include BMIs > 40 or BMI > 35 with any obesity-related disease [12]. There are several different bariatric surgery procedures (see Fig. 1), further outlined below. In the heart failure population specifically, bariatric surgery often times can also serve as a bridge to transplant or LVAD in those heart failure patients who struggle with qualifying for these advanced therapies based on BMI [33, 34].

### Types of bariatric surgery performed

The classification of bariatric surgeries falls into 3 broader categories: restrictive, malabsorptive, and combination [13]. The restrictive surgeries physically decrease stomach size, resulting in decreased food intake and earlier satiety; a couple examples of restrictive bariatric surgeries include sleeve gastrectomy and gastric banding. Sleeve gastrectomy consists of a single-day procedure that removes 75% of the stomach, leaving behind a narrow tube [14]. Gastric banding, on the other hand, does not permanently alter stomach structure, but rather places an adjustable band or silicone ring around the upper portion of the stomach to create a smaller stomach pouch [13, 14]. Both the sleeve gastrectomy and gastric banding can be performed laparoscopically [15].

The malabsorptive bariatric surgeries bypass segments of the GI tract so that less nutrient absorption may take place. One of the only bariatric surgeries to have only a malabsorptive mechanism is biliopancreatic diversion with or without duodenal switch. This procedure consists of removing a portion of the stomach and connecting it to a lower portion of the small intestines [16]. Unfortunately, this procedure is associated with long-term malabsorptive side effects such as decrease in absorption of essential nutrients.

Lastly, the combination procedures utilize both restrictive and malabsorptive mechanisms to achieve substantial weight loss. One such procedure is the Roux-en-Y gastric bypass. This procedure creates a new and much smaller stomach pouch, which is reconnected to the middle part of the small intestines (jejunum) and completely bypasses the upper portion of the small intestines (duodenum) [17]. Roux-en-Y gastric bypass currently is considered the “gold standard” and the most performed bariatric operation [13]. Additionally, Roux-en-Y is now performed laparoscopically to achieve a minimally invasive technique.

The benefits versus the potential complications of each of these types of bariatric surgeries are worthwhile comparing. While restrictive surgeries have the benefit of being single day procedures performed laparoscopically, they are often associated with the risk of developing cholelithiasis. Additionally, acute complications also include staple line leak both in the immediate post-surgical period (up to 5% of patients) and have been reported up to 8 years after intervention. Stricture formation can also occur in either an acute or delayed fashion, requiring invasive endoscopic dilation if bowel rest does not alleviate the stricture initially. Malabsorptive surgeries on the other hand, as the name implies, rely on decreased nutrient absorption as a means to achieve weight loss. As a result, it is no surprise that nutritional deficiencies occur, and this is the one of the most predominant complications. Additionally, long-term issues such as internal hernias may develop. As mentioned above, malabsorption occurs with less frequency in combination procedures such as Roux en Y bypass. Moreover, Roux en Y is the gold standard in weight loss surgery based on its durable long-term weight loss and improvement of obesity-related comorbidities. Albeit the bar for weight loss surgery, Roux en Y does not come without its own rare, but possible complications. Namely, the most frequent late complication is stomal stenosis occurring in about 5–15% of patients. Less frequently seen are bowel obstruction (1%), marginal ulcers (< 1%), and after open approaches, ventral hernia formation [18, 19].

### Pre-surgical care and run in period

The requirements to qualify for bariatric surgery follow guidelines as outlined by the National Institutes of Health. These include a BMI > 40 or BMI > 35 with one or more obesity-related disorders. Recently, the Medicare Coverage Advisory Committee has concluded that sufficient evidence exists to support these guidelines [20]. While many private health insurance carriers and Medicaid programs have followed the Advisory Committee’s lead, several loco-regional insurance companies have further required patients to complete a medical weight management program before procedure coverage approval [21]. The clinical impact of compliance to a mandatory pre-operative weight loss regimen, termed the pre-surgical care run in period, has remained controversial with inconsistency in the literature [22, 23]. Certain studies have suggested that these programs may not provide any additional benefit on long-term bariatric outcomes [24]. Rather, by mandating run in periods, payer companies are providing an obstacle to a procedure that may be both medically necessary and potentially life-saving [25, 26]. Meanwhile, other studies have shown that compliance with a multidisciplinary team prior to bariatric surgery protects against some post-operative complications [27]. It is important to bear in mind that conflicting results in the literature may be attributed to the relatively small patient populations included in studies as well as the heterogeneity of study protocols [23].

### Post-operative outcomes and safety

The safety and efficacy of bariatric surgery has been confirmed across many studies. In fact, several studies show that compared with non-surgical controls, patients who underwent bariatric surgery experienced a significantly larger weight loss and resolution or significant improvement in comorbidities such as diabetes, hyperlipidemia, hypertension, and obstructive sleep apnea [28]. The risks associated with bariatric surgery have been cited as very low, but with considerable variation between patent subgroups. In a prospective multicenter observational study of 30 day outcomes in 4776 patients undergoing bariatric surgical procedures at 10 clinical sites in the USA, these risks included but were not limited to death (0.3%), venous thromboembolism (0.4%), and re-intervention/operation at 30 days (3.1%), with 4.3% of patients having at least one major adverse outcome at 30 days [29].

## Existing literature

For the purposes of this review, Pubmed, Scopus, and Cochrane Library databases were searched for observational studies and randomized controlled trials focusing on patients with comorbid obesity and heart failure who have undergone bariatric surgery. A librarian’s assistance was used to develop a search algorithm that identified studies published in English inclusive of two patient populations: those with heart failure and those who met criteria for obesity. These search results were narrowed further to only include studies with at least one intervention being bariatric surgery. The following MeSH (Medical Subject Headings) terms and key words were used to identify heart failure: heart failure, cardiomyopathy(ies), cardiac failure, heart decompensation, myocardial failure, and myocardiopathy. In order to identify studies related to obesity, the following MeSH terms and keywords were used in the search queries: obesity, overweight, body mass index, waist circumference, and metabolic syndrome. Additionally, in order to identify studies that included bariatric surgery as the intervention, we used the following MeSH terms and keywords: bariatric, bariatric surgery, gastric bypass, gastroplasty, gastric band, gastrectomy, jejunoileal bypass, lipectomy, lipoabdominoplasty, biliopancreatic diversion, roux en y, lap band, and duodenal switch. The database search resulted in 862 initial studies identified. Eight were selected for inclusion in this review based on quality of study and pertinence.

Starting with the study done by McCloskey et al., the impact of bariatric surgery was evaluated in 14 patients. Ten patients underwent laparoscopic Roux-en-Y gastric bypass, 1 open Roux-en-Y gastric bypass, 2 sleeve gastrectomy, and 1 laparoscopic gastric banding. Preoperatively, two patients were characterized as NYHA class IV, six with class III, and six with class II heart failure. At 6 months’ follow-up, the mean BMI decreased by 14 kg/m^2^ (median excess BMI loss of 50.4%) and a mean improvement of LVEF from 23 to 32% and remained at a mean of 33% at follow-up all the way to 7 years. Of fourteen patients, eight had improvement in LVEF, two had no change in LVEF, two had a decrease in LVEF, and two had not yet undergone echocardiography at the end of the study. Improved LVEF correlated with improved functional capacity, as measured by NYHA classification. Postoperative NYHA classifications at 6 months’ follow-up consisted of two patients with classification III and twelve with classification II. Two patients went on to undergo successful cardiac transplantation.

With regard to patient safety, there were no deaths. Four patients had short-term complications observed up to 30-day post-surgery: pulmonary edema in one patient, transient hypotension in another, and transient renal insufficiency in two. In the initial six postoperative months, no patients required hospitalization related to heart failure compared with five admissions related to heart failure in the six months preceding bariatric surgery.

Ramani et al. performed a retrospective chart review of 12 patients with morbid obesity and systolic heart failure who underwent bariatric surgery and compared outcomes with 10 matched controls who were given diet and exercise counseling only. They showed that at median follow-up of 12 months, median BMI reduction was 53 to 38 kg/m^2^ in the group that underwent bariatric surgery (as compared with control group BMI 47 to 48 kg/m^2^). Additionally, their LVEF significantly improved from a mean of 22 to 35%, NYHA significantly improved from class 3 to 2.3, and their LV sizes were reduced. Clinically, their CHF admissions and use of diuretics were significantly reduced. In fact, 2 of the 12 patients were placed on the waiting list for heart transplant while 1 patient successfully underwent heart transplantation. One of the 12 bariatric surgery patients had transient pulmonary edema while another patient experienced acute renal failure. However, there were no reported post-operative myocardial infarctions or anastomotic leaks.

Miranda W.R. et al. looked at 19 patients with EF < 50% or class II-IV diastolic dysfunction and compared operative (roux-en-y) to non-operative outcomes retrospectively. The main focus of this study was to compare quality of life metrics between the two groups. There was a significant decrease in the operative groups BMI from a baseline of 55 to 35 kg/m^2^, and a 42% decrease in body weight. Clinically, these patients had significant improvement in resolution of their diabetes, better quality of life, and improvement in HF symptoms such as exertional dyspnea and lower extremity edema.

Lim et al.’s study included 7 patients with advanced systolic heart failure and LVEF < 25% who underwent bariatric surgery for durable weight loss in order to later undergo cardiac transplantation. The main postoperative outcomes were at median 406 days’ follow-up and involved a median decrease in BMI by 12.9 kg/m^2^ (median excess BMI loss of 64.4%) with a median improvement of LVEF from 20 to 30%. There was a markedly greater improvement in LVEF after laparoscopic sleeve gastrectomy compared with laparoscopic adjustable gastric band. At follow-up three patients were NYHA class III, one (LVAD patient) was class II, and three were class I, significant changes from pre-operative states. All patients lost enough weight to be considered eligible for cardiac transplantation. Two patients reported symptomatic improvement with little change in left ventricular function, meeting listing criteria. Three patients showed major improvement of LVEF and functional status, no longer requiring transplantation. As of the study’s publication, two patients went on to undergo successful cardiac transplantation. One patient, who underwent sleeve gastrectomy and partial fundoplication, required reoperation to correct postoperative esophageal and gastric ischemia caused by the fundoplication. The patient experienced acute kidney and liver dysfunction. No other patients experienced major perioperative complications. One patient was readmitted within 30 days of discharge due to pneumonia.

In Mikhalkova et al.’s observational study, 12 patients with HFpEF and obesity underwent echocardiography, magnetic resonance spectroscopy before, and 3 and 6 months after bariatric surgery. Over the course of the two follow-ups, several postoperative outcomes were noted. Patients progressively experienced fewer heart failure symptoms, as shown through decreased Minnesota Living with Heart Failure Questionnaire scores and NYHA classes. Patients also showed progressive weight loss and improvement in resting heart rate, with mean BMI decreasing by 13.2 kg/m^2^ and mean resting heart rate decreasing by 12 over 6 months. Left ventricular structure and function showed improvement after six months. There were decreases in left ventricular mass and relative wall thickness as well as improvements in left ventricular relaxation and left atrial filling pressure as measured by echocardiography. However, there was a non-significant decrease in ejection fraction. Cardiac fat was unchanged, while mean hepatic fat decreased to within normal limits. At 3 months’ follow-up, many plasma ceramides and sphingomyelins decreased, but these lipid changes did not correlate with changes in cardiac function.

In the study done by Punchai et al., 7 patients with end stage heart failure and LVAD therapy were included with the goal of increasing their candidacy for heart transplant. All of these patients were followed prospectively after bariatric surgery and the analysis showed positive outcomes after a mean follow-up period of 24 months—this included reductions in their BMI from 43.6 to 37.2 kg/m^2^, increase in LVEF from 19 to 22% and on average the NYHA class improvement from III to II. Additionally, 3 out of 7 patients underwent successful heart transplantations. Additional conclusions from the paper included identifying bariatric surgery as safe for patients with LVADs. While 5 out of the 7 patients experienced 30-day complications including GI bleeding, and acute cholecystitis complicated by septic shock, there were no mortalities at 1 year.

Moving on to the paper by Aleassa et al., 2810 patients with a principal discharge diagnosis of heart failure who had also had a history of prior bariatric surgery were identified and matched with those who had a principal diagnosis of heart failure but no history of bariatric surgery. The main postoperative outcomes assessed in this study were in-hospital mortality and length of stay after admission for heart failure exacerbation. There was a 50% reduction in in-hospital mortality among patients with a history of prior bariatric surgery compared with those without prior bariatric surgery. A 1-day mean reduction in length of stay was also observed among patients with a history of prior bariatric surgery compared with those without prior bariatric surgery—the supposition being that these results may be due to patients with prior bariatric surgery having less severe heart failure or fewer complications.

### Limitations of current studies

Of the 7 studies above (see Table 1), a range of limitations were identified—the most common limitation being small sample size, followed by short duration of follow-up, lack of generalizability as well as absence of identification of a pre-run in period or pre-surgical care.

**Table 1:** A range of limitations of the 7 studies (small sample size)

Title	Author	Year	Center	N	Patient population	Study design	Type of bariatric surgery performed	Follow-up time	Event rate	Baseline patient characteristics	Baseline EF	Post operative outcomes
Bariatric surgery improves cardiac function in morbidly obese patients with severe cardiomyopathy	McCloskey et al.	2007	Unspecified	14	14 patients with pre-op BMI > 40 + LVEF ≤ 35% who underwent bariatric surgery from 1998 - 2005	Retrospective study. Assessed short-term morbidity/mortality, length of stay, excess weight loss, pre- and post-op LVEF, NY Heart Association functional class	10 lapro Roux-en-Y, 1 open Roux-en-Y, 2 SG, 1 LGB	6 months	N/A	Patients with BMI > 40, EF ≤ 35%, who had undergone BS from 1998 to 2005	Improved from mean 23% to 32%	Mean decrease in BMI at 6 months of 14. Mean LVEF at 6 months significantly improved from 23 to 32% (*p* = 0.04), correlating with improved functional capacity (as measured by NYHA classification). Pre-op NYHA classification: 2 IV, 6 III, 6 II. 6 months post-op NYHA classification: 2 III, 12 II. Median length of stay was 3.0 days. Two patients underwent cardiac transplant after weight loss.
Safety and efficacy of bariatric surgery in morbidly obese patients with severe systolic heart failure	Ramani et al.	2008	University of Pittsburgh medical center	22	Queried the HF clinic database of U Pitt Med center for patients with advanced systolic HF and morbid obesity. (12). Identified 10 matched controls that did not undergo Bsx from 2001 to 2006	Retrospective study. Looked at obese patients with advanced HF and MO who underwent Bsx compared to matched controls that underwent conservative treatment (diet and exercise counseling)	laparoscopic Roux-en Y bypass, LSG, laparoscopic gastric banding, open Roux-en y bypass	1 year	1 pt had transient pulmonary edema. 1 pt had acute renal failure. No post-op Mis or anastomotic leaks.	BMI: 53, NYHA class 2.9 Matched controls: BMI 47.2, NYHA class 2.4	Bsx: LVEF 22% Control: LVEF 24%	Operative group, BMI decreased significantly (53 to 38, *p* < 0.01) while did not in control (47 to 48, *p* = 0.56), significant improvement in LVEF (22 to 35% *p* = 0.005; vs. 24 to 29% *p* ≥ 005.) and NYHA (3 to 2.3 (*p* = 0.02) vs. 2.4 to 3.3 *p* = 0.02) compared with controls, trending reduction in LV size, fewer CHF admissions (0.4 vs. 2.5 *p* = 0.04), reduction in diuretic use, 2 patients placed on waiting list for heart transplant, 1 patient successfully underwent
Impact of bariatric surgery on quality of life, functional capacity, and symptoms in patients with heart failure	Miranda et al.	2013	Mayo Clinic	19	849 patients referred for Roux-en-Y gastric bypass Residents of Olmsted County referred for BSx from 1990 to 2005	Retrospective. Non operative (6) vs. operative (13) in patients with HF based on a clinical diagnosis, EF < 50%, or grade II–IV diastolic dysfunction based on echocardiogram	Roux-en-Y	4.3 year for bariatric, 2.7 for non-operative	N/A	Bariatric group: 62, 146 kg, 55 BMI, EF 57% Non-bariatric group: 69, 132 kg, 42 BMI, EF 57.5%	Bariatric: 57% Non-bariatric: 57.5%	*t* median follow-up of 406 days, median BMI reduction was 12.9 kg/m^2^ (*p* = 0.017), post-op LVEF improved to median 30% (*p* = 0.039) from median pre-op LVEF 20.0%. Pre-op NYHA: 6 III, 1 II. Post-op NYHA: 3 I, 3 III, 1 LVAD patient improved to II. 2 patients
Bariatric Surgery Provides a "Bridge to Transplant" for Morbidly Obese Patients with Advanced Heart Failure and May Obviate the Need for Transplantation	Lim et al.	2016	St Vincent's Hospital, Sydney, Australia	7	7 patients with advanced systolic heart failure and LVEF < 25% who underwent bariatric surgery for durable weight loss in order to later undergo cardiac transplantation	Retrospective single-center cohort analysis. Collected demographic data, clinical characteristics, heart failure etiology, preoperative LVEF and New York Heart Association (NYHA) functional class, type of bariatric surgery performed, and intraoperative right heart catheterization data. Assessed efficacy of surgery with absolute weight loss and BMI reduction, LVEF, and NYHA class at a minimum of 6 months post surgery.	4 laparoscopic sleeve gastrectomy (LSG), 3 laparoscopic adjustable gastric band (LAGB)	Median 406 days	N/A	Patients with advanced systolic HF and LVEF < 25% who underwent BS at St. Vincent's Hospital, Sydney, between Jan 2009 and Sep 2014	Improved to median of 30%	At median follow-up of 406 days, median BMI reduction was 12.9 kg/m^2^ (*p* = 0.017), post-op LVEF improved to median 30% (*p* = 0.039) from median pre-op LVEF 20.0%. Pre-op NYHA: 6 III, 1 II. Post-op NYHA: 3 I, 3 III, 1 LVAD patient improved to II. Two patients underwent successful cardiac transplant. Two patients reported symptomatic improvement w/ little change in LV function, successfully meeting listing criteria. Three patients showed marked improvement of LVEF and functional status, removing requirement for transplantation
Bariatric surgery–induced cardiac and lipidomic changes in obesity-related heart failure with preserved ejection fraction	Mikhalkova et al.	2018	Washington University School of Medicine surgery center	12	12 women with HFpEF and obesity (BMI > 35)	Prospective study. Used labs, HF questionnaires, magnetic resonance spectroscopy, echocardiography, and mass spectrometry to assess HF symptoms, diastolic function, myocardial fat deposition, and plasma ceramides/sphingolipids. A validation cohort (*n* = 22) also underwent gastric bypass surgery, and their plasma lipidomic changes were assessed	Roux-en-Y	3 months, 6 months	N/A	Women 35–65 years old with BMI > 35, diagnosis of HFpEF. EXCLUDE current tobacco use, an inability to be ambulatory or to lie flat for procedures, pregnancy or lactation, cardiac conditions that interfered with assessment of diastolic function (e.g., constrictive pericarditis or atrial fibrillation/flutter), contraindication to magnetic resonance spectroscopy, other major systemic disease except for type 2 diabetes, ejection fraction < 50%, uncontrolled hypertension, significant pulmonary hypertension by history and/or echocardiography, and/or evidence of ischemia on screening stress echocardiogram	N/A	HF questionnaire scores, cardiac mass, and liver fat decreased; early relaxation at septal + lateral mitral value annuli increased; cardiac fat unchanged. Decreased plasma ceramide/sphingolipid, but plasma lipid + cardiac function changes did not correlate
Laparoscopic sleeve gastrectomy in heart failure patients with left ventricular assist device	Punchai et al.	2019	Cleveland Clinic	7	(7) Patients with ESHF NYHA class 2-4 who underwent LVAD implantation and subsequent Bsx from 2013 to 2017.	Retrospective study. Compared pre and post LSG in obese patients with ESHF with LVAD	LSG	Mean 24 months	5 pts (71%) experienced 30-day complications, including GI bleeding, acute cholecystitis with septic shock. No 1 year mortality, arrhythmias, UTIs, nausea.	Median age: 39 years Median BMI: 43.6	Pre-op 17%	The LVEF increased (19 to 22%), the NYHA class improved (3 to 2), 3 patients underwent successful heart transplantations, BMI decreased (43.6 to 37.2)
Impact of bariatric surgery on heart failure mortality	Aleassa et al.	2019	Healthcare cost and utilization project nationwide inpatient sample (2007–2014)	2810	2810 patients with principal discharge diagnosis of HF who also had a history of prior bariatric surgery	Retrospective analysis. Patients were matched 1:5 with patients who had similar principal diagnoses but no history of bariatric surgery (control group-1 had BMI ≥ 35, control group-2 was not limited to patients with obesity). Used multivariate regression models to calculate odds ratio and 95% confidence interval of mortality and length of stay	Unspecified	N/A	In-hospital mortality rates: bariatric surgery group vs control group-1 (0.96% vs 1.86%, OR 0.52, 95% CI 0.35-0.77, p=0.0013), bariatric surgery group vs control group-2 (0.96% vs 1.86%, OR 0.52, 95% CI 0.35-0.77, *p* = 0.0011). LOS: bariatric surgery group vs control group-1 (4.8 ± 4.4 vs 5.7 ± 5.7 days, *p* < 0.001), bariatric surgery group vs control group-2 (4.8 ± 4.4 vs 5.4 ± 6.3 days, *p* < 0.001)	All patients 18+ years old with primary admission diagnosis of HF. Case group included all of these patients with hx of BS. Control group-1 included patients with no hx of BS with BMI 35+. Control group-2 included patients with no hx of BS + not exclusively with obesity	N/A	In-hopsital mortality rates after admission for HF were significantly lower in patients with history of bariatric surgery compared with both control groups. LOS was also significantly shorter in bariatric surgery group compared with both control groups

In addition to small sample size, Mikhalkova’s study identifies short duration of follow-up a shortcoming as well, and poor generalizability with an inability to extend their findings to men or other subjects that were not in the inclusion criteria. As with many bariatric surgery studies, women were chosen on the basis that they made up a vast majority of subjects that underwent gastric bypass surgery. In the study done by McCloskey et al., limitations included interpretation of echocardiograms by different physicians at the same institution, introducing a degree of subjective interpretation variability; specifically 1 patient’s LVEF was determined not by echocardiography but by catheterization. Additionally, there was no differentiation made between ischemic and non-ischemic cardiomyopathy in the subjects that were selected clinically, which brings up the limitation of generalizability as well. Similarly, Punchai et al.’s study consisted of a small sample, and while median follow-up was 24 months, one patient only had a 2-month follow-up further limiting consistency among follow-up. Ramani et al. comment that their study group was too small and examined retrospectively with poor generalizability as the majority of their patients included had non-ischemic cardiomyopathy. HFpEF patients were furthermore excluded, and their control group had a statistically significant difference in baseline BMI, with selection bias coming into question.

Of all the studies, only 2 mentioned pre-surgical care and run in periods. Punchai et al. mentions having all patients assessed by a multidisciplinary team before surgery—further details were not given about pre surgical care in this study. Lim et al. describes a run in period that included having a calorie restricted eating plan, but nothing more. The rest of the studies did not include any detail or suggestion of pre surgical care.

## Future directions

Both obesity and heart failure pose major growing health and economic burdens worldwide. The connection between the conditions remains an area of interest for several reasons. One of the main comorbidities of obesity is heart failure, with obese individuals having double the risk of heart failure as compared with individuals with normal weight, and obesity has been shown to be an independent risk factor for morbidity and mortality in heart disease. From ventricular remodeling to hormonal changes, several interconnecting systems play a role in the relationship of obesity and heart failure. This makes the concept of using bariatric surgery to improve cardiac function, enable for LVAD candidacy or even heart transplant intriguing.

A limited number of mostly retrospective studies have been done that show improved cardiac function and outcomes in patients with heart failure that undergo bariatric surgery when compared with those who did not. Limitations are similar in most of these studies—small population size, generalizability of data produced, lack of details about pre surgical care, and subjectivity of reporting of cardiac function when using modalities such as echocardiography. Future directions include designing prospective studies with a larger patient population as well as using modalities such as MUGA scans to obtain more objective measures of cardiac function and ejection fraction. Nonetheless, it is reasonable to say that using bariatric surgery as a means to improve outcomes in patients with heart failure at the levels of cardiac function, quality of life, and to increase candidacy for heart transplant is an under-utilized intervention that should perhaps be more often implemented as part of the treatment protocols of heart failure patients.

## Abbreviations

HF: Heart failure
HFrEF: Heart failure reduced ejection fraction
HFpEF: Heart failure preserved ejection fraction
HTN: Hypertension
LV: Left ventricle
LVEF: Left ventricular ejection fraction
BMI: Body mass index
MUGA: Multi-gated acquisition
